# Oral Administration of Universal Bacterium-Vectored Nucleocapsid-Expressing COVID-19 Vaccine is Efficacious in Hamsters

**DOI:** 10.1128/spectrum.05035-22

**Published:** 2023-03-14

**Authors:** Qingmei Jia, Helle Bielefeldt-Ohmann, Rachel M. Maison, Airn Hartwig, Saša Masleša-Galić, Richard A. Bowen, Marcus A. Horwitz

**Affiliations:** a Division of Infectious Diseases, Department of Medicine, Center for Health Sciences, School of Medicine, University of California Los Angeles, Los Angeles, California, USA; b Australian Infectious Diseases Research Centre, University of Queensland, St. Lucia, Queensland, Australia; c Department of Biomedical Sciences, Colorado State University, Fort Collins, Colorado, USA; Institute of Microbiology, Chinese Academy of Sciences

**Keywords:** vaccine, COVID-19, SARS-CoV-2, oral administration, single vector platform vaccine, vaccine vector, membrane protein, mouse, nucleocapsid protein, single vector platform, Syrian hamster, bacterial vector, oral vaccine

## Abstract

Oral delivery of an inexpensive COVID-19 (coronavirus disease 2019) vaccine could dramatically improve immunization rates, especially in low- and middle-income countries. Previously, we described a potential universal COVID-19 vaccine, rLVS Δ*capB*/MN, comprising a replicating bacterial vector, LVS (live vaccine strain) Δ*capB*, expressing the highly conserved SARS-CoV-2 (severe acute respiratory syndrome coronavirus 2) membrane and nucleocapsid (N) proteins, which, when administered intradermally or intranasally, protects hamsters from severe COVID-19-like disease after high-dose SARS-CoV-2 respiratory challenge. Here, we show that oral administration of the vaccine also protects against high-dose SARS-CoV-2 respiratory challenge; its protection is comparable to that of intradermal, intranasal, or subcutaneous administration. Hamsters were protected against severe weight loss and lung pathology and had reduced oropharyngeal and lung virus titers. Protection against weight loss and histopathology by the vaccine, which in mice induces splenic and lung cell interferon gamma in response to N protein stimulation, was correlated in hamsters with pre-challenge serum anti-N TH1-biased IgG (IgG2/3). Thus, rLVS Δ*capB*/MN has potential as an oral universal COVID-19 vaccine.

**IMPORTANCE** The COVID-19 pandemic continues to rage into its fourth year worldwide. To protect the world’s population most effectively from severe disease, hospitalization, and death, a vaccine is needed that is resistant to rapidly emerging viral variants of the causative agent SARS-CoV-2, inexpensive to manufacture, store, and transport, and easy to administer. Ideally, such a vaccine would be capable of oral administration, especially in resource-poor countries of the world where there are shortages of needles, syringes and trained personnel to administer injectable vaccines. Here, we show that oral administration of a bacterium-vectored vaccine meeting all these criteria protects naturally susceptible Syrian hamsters from severe COVID-19-like disease, including severe weight loss and lung pathology, after high-dose SARS-CoV-2 respiratory challenge. As the vaccine is based upon inducing immunity to highly conserved SARS-CoV-2 membrane and nucleocapsid proteins, as opposed to the rapidly mutating Spike protein, it should remain resistant to newly emerging SARS-CoV-2 variants.

## INTRODUCTION

The coronavirus disease 2019 (COVID-19) pandemic, caused by severe acute respiratory syndrome coronavirus 2 (SARS-CoV-2), continues to rage into its fourth year worldwide. As of this writing, cases exceed 585 million and deaths exceed 6.4 million (1). Approximately 60% of the world’s population has been vaccinated against COVID-19, although vaccination rates vary widely and are notably low throughout Africa (2).

While current vaccines have been highly successful in diminishing symptomatic infection, hospitalization, and death, SARS-CoV-2 variants are continuously emerging which render these vaccines, nearly all of which are centered on inducing neutralizing antibodies to the Spike (S) protein, less and less effective (3). One approach to rectifying this problem is redesigning and testing new vaccines based on currently circulating major SARS-CoV-2 variants; however, by the time such vaccines are ready for distribution, new variants have already or are soon likely to emerge (3–5). Hence, it has become increasingly clear that to combat the COVID-19 pandemic most effectively, we need universal vaccines that are resistant to virus escape mutations.

The optimal COVID-19 vaccines would be safe, potent, and affordable, as well as universal. Our rLVS Δ*capB*/MN (MN) vaccine, comprising a replicating bacterial vector expressing the SARS-CoV-2 membrane (M) and nucleocapsid (N) proteins, appears to fulfill these criteria (6). With respect to safety, the vaccine vector (LVS Δ*capB*) (7) is a further attenuated version (>10,000-fold less virulent in a sensitive mouse model) of a tularemia vaccine (LVS, live vaccine strain) that has been administered to millions of people (8–11); LVS itself was highly attenuated (two major attenuating deletions and several minor ones) from a relatively low-virulence subspecies (subsp. *holarctica*) of Francisella tularensis, the agent of tularemia. With respect to potency, two immunizations of the MN vaccine intradermally (ID) or intranasally (IN) have been demonstrated to protect golden Syrian hamsters from COVID-19-like disease after high-dose SARS-CoV-2 respiratory challenge (6). With respect to affordability, the vaccine can be grown to hundreds of millions of doses overnight in simple broth culture without the need for extensive purification, and after lyophilization, stored and transported at refrigerator temperature. Finally, with respect to universality, while most approaches to a universal vaccine have focused on inducing broadly neutralizing antibodies to the S protein or its receptor-binding domain (12), the MN vaccine is centered on inducing immunoprotective humoral and T cell responses to highly conserved SARS-CoV-2 proteins, particularly the M and N proteins. By way of comparison, at least 35 mutations have been identified in the S protein versus 3 mutations/1 deletion in the N protein in the current Omicron variant of concern (13, 14). Because T cell immunity entails recognition of different parts of these immunoprotective antigens by different MHC (major histocompatibility complex) types, and these antigens are highly conserved in any case, this vaccine should be highly resistant to escape mutations. In support of a key role for T cell immunity, studies have shown that T cells can contribute to clearance of SARS-CoV-2 (15) and that pre-existing memory T cells cross-reactive with a SARS-CoV-2 conserved protein can rapidly expand and protect against COVID-19 (16). Moreover, non-neutralizing antibody to the N protein, which has been shown to be displayed on the surface of SARS-CoV-2 infected cells, may play an important role in immunoprotection via antibody-dependent cellular cytotoxicity (ADCC) (17).

The ideal COVID-19 vaccines would additionally be capable of oral administration, as this would address two major factors hampering more all-inclusive vaccination. First, a significant contributing factor to poor vaccination rates in low-income countries is lack of needles, syringes, and most importantly, trained personnel to deliver an injectable vaccine (18). A vaccine capable of oral delivery would eliminate this factor. Second, a contributing, albeit difficult to quantitate, factor to vaccine hesitancy everywhere is the fear of needles, which also would be rendered moot by an oral vaccine.

Here, we describe a study of the efficacy of oral delivery of the MN vaccine in golden Syrian hamsters, which develop COVID-19-like disease after high-dose respiratory challenge with SARS-CoV-2, including severe weight loss and substantial pulmonary pathology similar to that which develops in humans. We show that oral administration of the vaccine lowers oropharyngeal and lung titers of SARS-CoV-2 and protects hamsters from severe weight loss and lung pathology.

## RESULTS

### Optimization of oral vaccination regimen.

**FIG 1:**
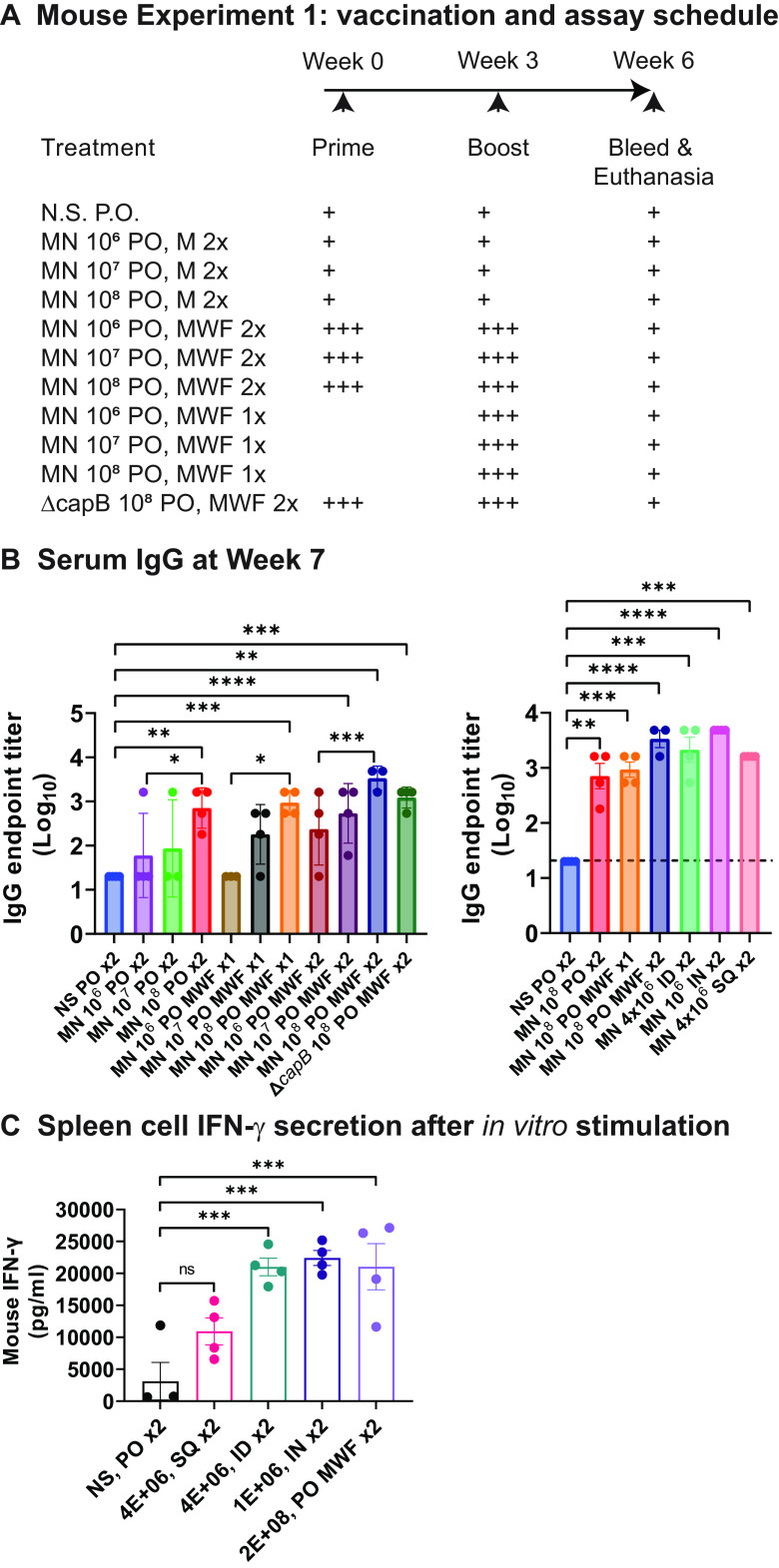
Antibody and T cell-mediated responses in mice to heat-inactivated live virus strain (HI-LVS) induced by the rLVS Δ*capB*::MN vaccine administered orally is dose-dependent, and the antibody response induced by the highest oral (PO) dose is comparable to that induced by the vaccine administered intradermally, intranasally and subcutaneously. (A) Experiment schedule. BALB/c mice, 4/group, were immunized at week 0 and week 3 orally by gavage once a week (Monday, M) or three times a week (Monday, Wednesday, Friday, MWF) or immunized intradermally (ID), intranasally (IN), or subcutaneously (SQ) once a week (Monday) with normal saline (NS) or three escalating doses (10^6^, 10^7^, and 10^8^) of rLVS Δ*capB*::MN (MN, membrane and nucleocapsid) vaccine. On week 6, all mice were bled and euthanized. (B) Serum IgG antibody. Sera were assayed for anti-HI-LVS antibody. Antibody endpoint titer is expressed as log_10_ the reciprocal of the highest serum dilution that is a minimum of 0.05 optical density units above the mean of the sham-immunized (NS) control serum plus 3 standard deviations at the same dilution. Data are mean log_10_ endpoint titer ± standard error (SE). *, *P < *0.05; **, *P < *0.01; ***, *P < *0.001; ****, *P < *0.0001 by one-way analysis of variance (ANOVA) with Tukey’s multiple-comparison test (GraphPad Prism version 9.2.0). (C) Splenocyte interferon gamma (IFN-γ) secretion. Splenocytes were stimulated with HI-LVS for 6 days, and supernatant was assayed for mouse IFN-γ. ***, *P* < 0.001, by one-way ANOVA with Tukey’s test (GraphPad Prism 9.2.0).

Oral delivery of a highly acid-sensitive live vaccine to small animals poses a major practical challenge because, unlike with humans, it is not easy to administer the vaccine in a capsule designed to protect the vaccine from stomach acid en route to the preferred release site in the intestine. For the purpose of this proof-of-concept study, our approach to this challenge was to develop a regimen for oral administration that involves briefly neutralizing stomach acid prior to vaccine delivery. In a preliminary mouse study, we established an oral immunization regimen by evaluating antibody responses induced by a rLVS Δ*capB* platform MN vaccine administered orally at different doses and frequencies and compared the antibody responses induced by the vaccine administered orally (PO), intradermally (ID), intranasally (IN), or subcutaneously (SQ). Because antibody responses in BALB/c mice to the nucleocapsid (N) protein are poor, we focused on antibody responses to the vector by assaying antibody to heat-inactivated (HI)-LVS. We immunized groups of 4 BALB/c mice PO by gavage once (Monday [M]) or three times a week (Monday, Wednesday, Friday [MWF]) at week 0 and week 3 with normal saline (NS) or three escalating doses (10^6^, 10^7^, 10^8^) of the rLVS Δ*capB*::MN vaccine, an antibiotic resistance marker-free version of the vaccine that expresses the MN proteins from the chromosome; mice immunized PO with the LVS Δ*capB* vector served as controls. Thirty minutes prior to PO immunization, we administered 0.1 mL of 10% (wt/vol) sodium bicarbonate to the animals by gavage to neutralize gastric acid. Mice immunized with the MN vaccine ID, IN, or SQ served as controls for PO administration. On week 6, all the animals were anesthetized, bled, and euthanized, and their spleens and lungs were removed (Fig. 1A). We assayed their sera for IgG antibody specific to HI-LVS. As shown in Fig. 1B (left graph), the anti-HI-LVS IgG antibody titer induced by the vaccine administered PO is vaccine dose-dependent; vaccines administered three times a week (MWF) and boosted similarly 3 weeks later induced a higher level of antibody than vaccines administered once a week and boosted 3 weeks later. Importantly, as shown in Fig. 1B (right graph), the anti-HI-LVS IgG antibody titer induced by the vaccine administered with the highest PO dose is comparable to that induced by the vaccine administered ID, IN, and SQ. Additionally, we assessed the capacity of the vaccine to induce a T cell-mediated immune response by the various administration routes. Of note, administration of the vaccine twice to BALB/c mice by all four routes induces splenic lymphocyte production of interferon gamma (IFN-γ) in response to HI-LVS; differences from sham mice were significant for administration by the PO, ID, and IN routes (*P < *0.001) (Fig. 1C).

Subsequently, we compared antibody responses to the rLVS Δ*capB*/MN vaccine, which expresses the MN proteins from a plasmid, in BALB/c mice after three immunizations PO, IN, and ID. We immunized groups of 4 BALB/c mice three times, 3 weeks apart, with rLVS Δ*capB*/MN administered ID, IN, or PO (MWF) as illustrated in Fig. S1A (supplemental material). At week 8, the mice were anesthetized and bled, and their sera assayed for IgG antibody-specific HI-LVS and additionally to SARS-CoV-2 N and M protein. As shown in Fig. S1B, mice immunized PO with rLVS Δ*capB*/MN produced high titers of IgG antibody to HI-LVS. Anti-HI-LVS antibody levels in mice immunized PO, ID, or IN were comparably high and all significantly greater than those in unvaccinated mice (*P < *0.0001). Previously, we observed that in BALB/c mice, antibody responses to the N and M protein were poor when the rLVS Δ*capB*::MN vaccine was given once or twice. However, ID immunization three times with the rLVS Δ*capB*/MN vaccine induced a significantly elevated anti-N titer compared to that in unvaccinated mice. PO administration also induced an elevated anti-N IgG titer comparable to that of ID immunization, and significantly greater than that in unvaccinated mice (Fig. S1B). PO administration also induced an elevated anti-HI-LVS IgG titer significantly greater than that in unvaccinated mice and that of ID immunization (Fig. S1B).

While protection against COVID-19-like disease in hamsters is correlated with anti-N IgG antibody, protection is likely also mediated by T cells. Consistent with this, as shown in Fig. S2, lung cells from mice immunized PO with LVS Δ*capB*/MN produced greater amounts of IFN-γ in their lungs in response to N protein or N protein peptides than unvaccinated mice (Fig. S2A). Levels of IFN-γ produced by spleen cells of immunized mice were not significantly different from those in control unvaccinated mice (Fig. S2B). In mice immunized PO with the MN vaccine, the levels of IFN-γ produced by lung (Fig. S2C) and spleen (Fig. S2D) cells after *in vitro* stimulation with HI-LVS were comparable to levels produced by cells from mice immunized ID and IN. PO administration also induced significantly greater level of anti-HI-LVS IFN-γ production than that of unvaccinated mice in the spleen (Fig. S2D). Thus, PO administration induces SARS-CoV-2 and LVS Δ*capB* vector-specific T cell-mediated immune responses.

Thus, we chose the rLVS Δ*capB*/MN vaccine administered PO three times to evaluate the efficacy of the vaccine in the Golden Syrian hamster model.

### MN vaccine administered orally protects against weight loss after SARS-CoV-2 respiratory challenge in golden Syrian hamsters.

**FIG 2:**
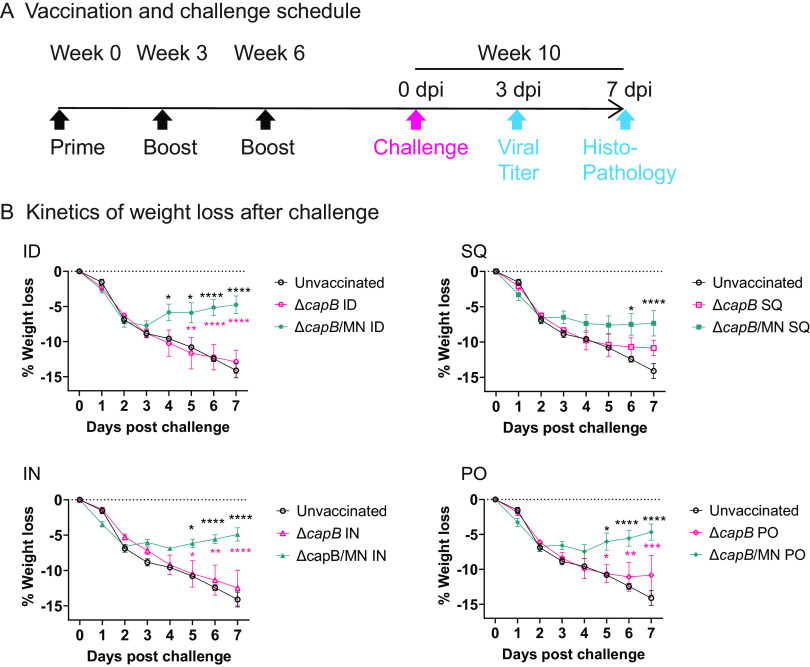
Experimental schedule and weight loss after challenge. (A) Experiment schedule. Syrian hamsters (8/group, 4 females, 4 males) were immunized ID, IN, SQ, or PO three times on weeks 0, 3, and 6 with LVS Δ*capB* vector (Δ*capB*) or rLVS Δ*capB/*MN (Δ*capB*/MN) vaccine; challenged IN on week 10 with 10^4^ PFU of SARS-CoV-2 (2019-nCoV/USA-WA1/2020 strain); and monitored closely daily for clinical signs of infection, including weight loss. Half of the animals were euthanized for lung viral titers at 3 days post challenge (dpi); the other half were euthanized for lung histopathology at 7 dpi. (B) Weight change after challenge. From days 0 to 3, *n* = 8/group; from days 4 to 7, *n* = 4/group. Data are mean percent weight loss ± standard deviation. Mean % changes were compared among groups on each day using a repeated measure (mixed) analysis of variance model since observations on the same animal over days are correlated. *P* values for comparing mean changes were determined to be significant using Tukey’s adjusted criterion: *, *P < *0.05; **, *P < *0.01; ***, *P < *0.001; ****, *P < *0.0001 (black asterisks, versus unvaccinated; red asterisks, versus vector control). Normal quantile plot examination of the residual errors and the Shapiro-Wilk test (W = 0.984) confirmed that the data followed a normal distribution, allowing the use of a parametric model.

To examine the protective efficacy of the MN vaccine administered PO in hamsters, a natural model of severe COVID-19-like disease, we immunized the animals (8/group) ID, SQ, IN, or PO 3 times, 3 weeks apart, with the MN vaccine, challenged them at week 10 with 10^4^ PFU SARS-CoV-2 (2019-nCov/USA-WA1/2020 strain), and monitored them closely for weight changes. Half of the animals were euthanized on day 3 post-challenge to assay lung viral titers and half of the animals were euthanized on day 7 post-challenge to assay lung histopathological changes (Fig. 2A). Hamsters immunized PO were administered the vaccine 3 times (MWF) per week. As shown in Fig. 2B, hamsters immunized ID, SQ, IN, or PO with the MN vaccine were significantly protected against severe weight loss after high-dose SARS-CoV-2 IN challenge on days 5 to 7 post-challenge (*P *< 0.01 to 0.0001). All animals lost approximately 5% to 7% of their total body weight during the first 2 days after challenge, in part due to being anesthetized with ketamine-xylazine for the challenge; however, hamsters immunized with the MN vaccine ID, IN, or PO rapidly began to recover from the weight loss starting on day 3 or 4 post-challenge, such that hamsters immunized ID, IN, or PO regained 36%, 52%, and 41% (mean ± standard error [SE]: 43% ± 4%) of the lost weight by day 7, significantly different from unvaccinated animals (*P < *0.0001 versus each MN-vaccinated group). In hamsters immunized SQ, body weight plateaued on day 3 such that by day 7, the difference in weight loss between this group and unvaccinated hamsters was also highly significant (*P < *0.0001). The % weight loss for hamsters immunized PO, IN, and ID was also significantly less than that of animals immunized with the vector control by the same route (*P < *0.001 to 0.0001). Unvaccinated animals continued to lose weight after challenge until they were euthanized on day 7, by which time they had lost a mean of 14% of their total body weight. Animals immunized with the vector control lost slightly less weight than unvaccinated animals (differences not significant); this may have reflected a small beneficial nonspecific immunologic effect, as has been hypothesized for BCG (bacillus Calmette-Guérin) and other vaccines (19–22).

### MN vaccine administered orally protects against severe lung pathology in SARS-CoV-2-challenged hamsters.

**FIG 3:**
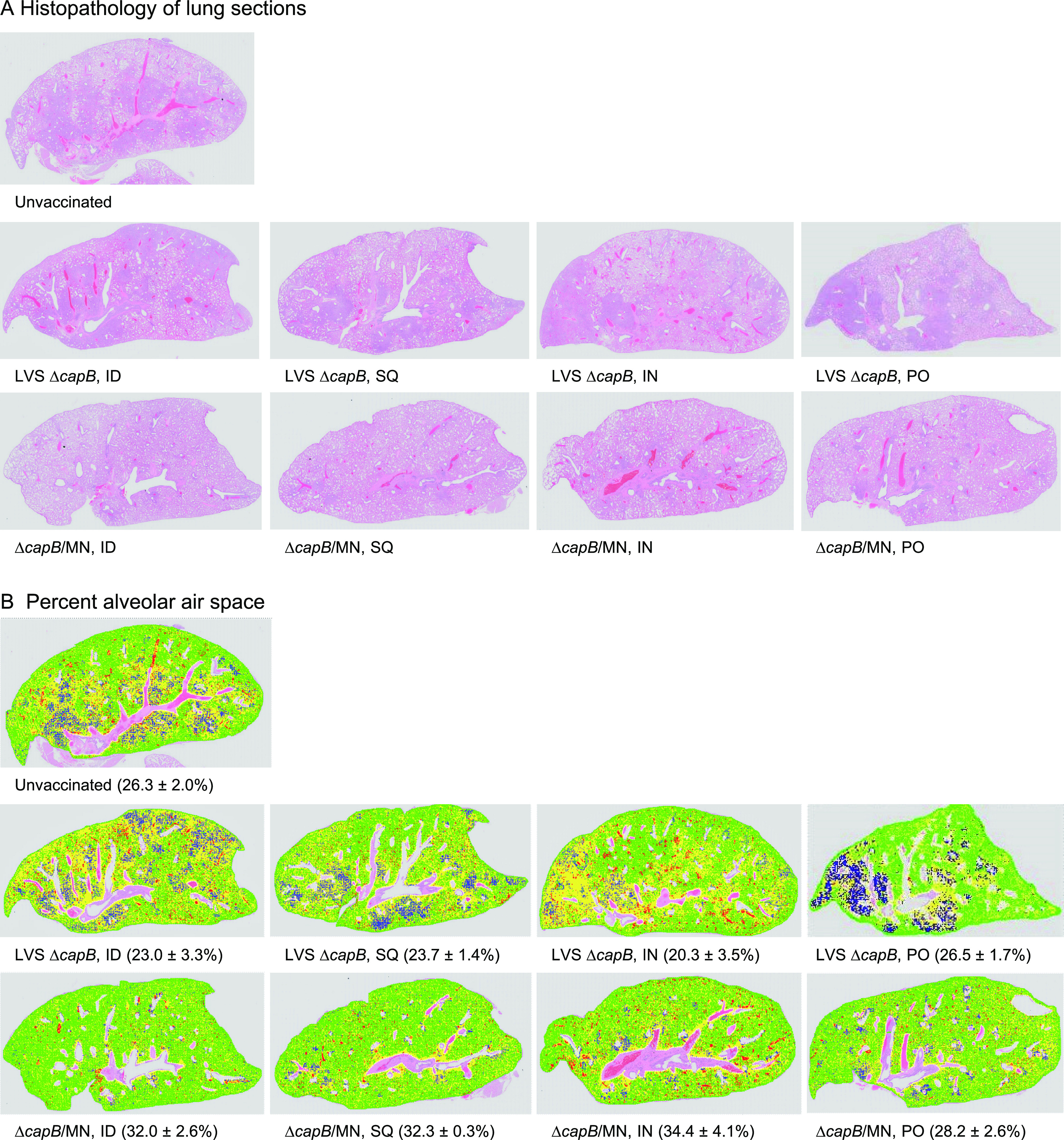
Lung histopathology and percent alveolar air space. Hamsters were not immunized or immunized ID, SQ, IN, or PO with LVS Δ*capB* vector (Δ*capB*) or the MN (Δ*capB*/MN) vaccine as described in Fig. 2A and euthanized at 7 days post-challenge for histopathological examination of lungs. (A) Histopathology (hematoxylin and eosin [H&E]-stained lung sections). (B) Mean percent alveolar air space. Mean ± SE are shown beneath each panel.

**FIG 4:**
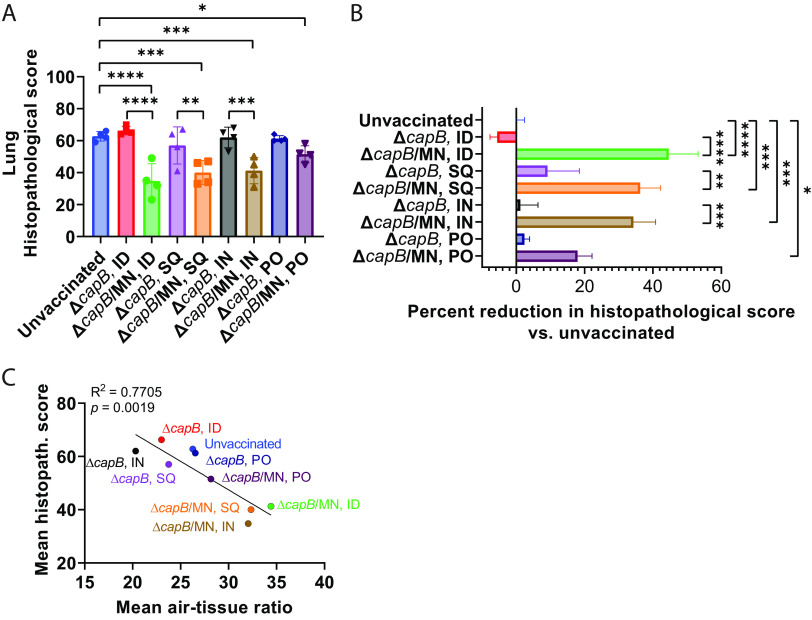
Correlation between lung histopathological score and percent alveolar air space on day 7 after SARS-CoV2 intranasal challenge. Hamsters were not immunized or immunized ID, SQ, IN, or PO with LVS Δ*capB* vector (Δ*capB*) or the MN (Δ*capB*/MN) vaccine as described in Fig. 2A and euthanized at 7 days post-challenge for histopathological examination of lungs. Left and right cranial and caudal lung histopathology were separately scored on a scale of 0 to 5 or 0 to 4 for overall lesion extent, bronchitis, alveolitis, pneumocyte hyperplasia, vasculitis, and interstitial inflammation, and the scores for each lung segment were summed for each animal. Histopathological score evaluation was performed by a single pathologist blinded to the identity of the groups. Each symbol represents one animal. (A) Total histopathological scores. (B) Percentage reduction of total lung pathological score compared with unvaccinated animals. (C) Correlation between lung histopathological score and percent alveolar air space. Data are mean ± SE. *, *P < *0.05; **, *P < *0.01; ***, *P < *0.001; ****, *P < *0.0001 by LSD test (GraphPad Prism 9.2.0).

To evaluate vaccine efficacy against SARS-CoV-2-induced lung disease, we assessed left and right lung cranial and caudal histopathology on day 7 post-challenge, which peaks in unvaccinated animals at this time point (6, 23). As shown in Fig. 3A, Fig. S3, Fig. 4A and B, and Table S1, hamsters immunized ID, SQ, IN, or PO with the MN vaccine were consistently protected against severe lung pathology after SARS-CoV-2 IN challenge. Consistent with the histopathological assessment, hamsters immunized with the MN vaccine had a greater percentage of alveolar air space than unvaccinated hamsters or hamsters vaccinated with the LVS Δ*capB* vector control (Fig. 3B), although for the PO groups, differences in percent air space between the MN-vaccinated and the vector control groups did not reach statistical significance. The lung histopathological scores in the MN-vaccinated groups were significantly lower than those of unvaccinated animals and animals vaccinated with the LVS Δ*capB* vector via the same route (*P < *0.0001 for ID, *P < *0.001 for SQ and IN, and *P < *0.05 for PO versus unvaccinated hamsters; *P < *0.0001 for ID, *P < *0.01 for SQ, and *P < *0.001 for IN versus vector control when administered by the same route) (Fig. 4A). Compared with unvaccinated hamsters, the total histopathological score in the cranial and caudal lungs of the MN-immunized hamsters was reduced, on average, by 45% when the vaccine was administered ID (*P < *0.0001), 36% when the vaccine was administered SQ (*P < *0.001); 34% when the vaccine was administered IN (*P < *0.001), and 18% when the vaccine was administered PO (*P < *0.05) (Fig. 4B). The mean percent alveolar air space correlated negatively with the mean lung histopathological score (*R*^2^ = 0.7705, *P = *0.0019) (Fig. 4C).

### MN vaccine administered orally protects against SARS-CoV-2 viral replication in the oropharynx and lungs in hamsters.

**FIG 5:**
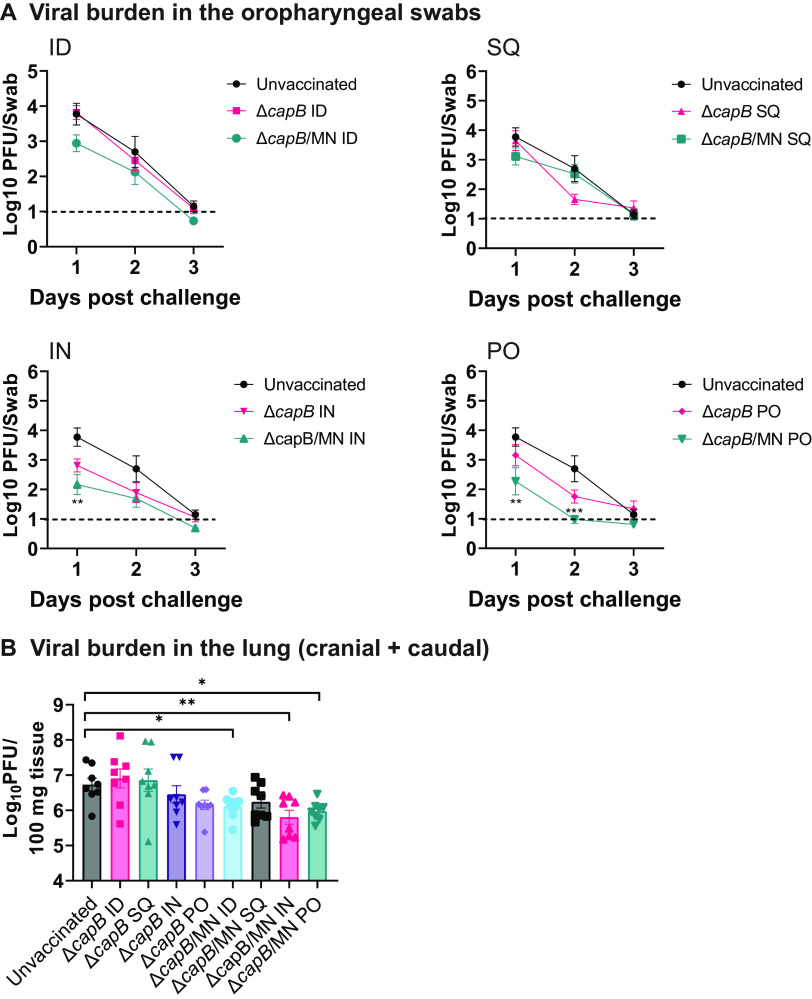
Viral load in oropharyngeal swabs and lung. Hamsters were immunized and challenged as described in Fig. 2A. (A) Oropharyngeal swabs were collected from all animals at 1, 2, and 3 days post-challenge and assayed for viral load by plaque assay. Data are mean log_10_ PFU per mL. **, *P < *0.01; ***, *P < *0.001 (color-coded to each vaccine) versus unvaccinated hamsters by ANOVA with Tukey’s *post hoc* comparisons test (GraphPad Prism 9.2.0). Dotted line: limit of detection. (B) Lungs (caudal + cranial) were homogenized, and viral burden assayed by plaque assay. Values are mean ± SE. *, *P < *0.05; **, *P < *0.01 by one-way ANOVA with LSD multiple-comparisons test (GraphPad, Prism 9.2.0).

We assayed viral burdens after respiratory challenge with SARS-CoV-2 in oropharyngeal swabs obtained daily at 1 to 3 days post-challenge and in the lungs at 7 days post-challenge. With regard to oropharyngeal swab plaque forming units (PFU), as shown in Fig. 5A, hamsters vaccinated with the MN vaccine PO had significantly lower PFU than unvaccinated animals on day 1 (*P < *0.01) and day 2 (*P < *0 0.001) post-challenge. Hamsters vaccinated with the MN vaccine ID and IN also had lower PFU than the unvaccinated animals on days 1 and 2 post-challenge, but the difference was statistically significant only for IN vaccinated hamsters on day 1 post-challenge (*P < *0.01). Hamsters vaccinated with the MN vaccine SQ showed a small reduction in PFU on day 1 which was not statistically significant.

With regard to lung PFU, as shown in Fig. 5B, hamsters vaccinated with the MN vaccine by all routes had lower PFU in their lungs (caudal + cranial) than the unvaccinated animals, and the differences were statistically significant for hamsters immunized PO (*P < *0.05), ID (*P < *0.05), and IN (*P < *0.01).

This result is consistent with our previous finding that the MN vaccine administered ID or IN protects against weight loss, severe histopathology in the lung, and viral loads in the oropharynx and lung (6). Thus, the MN vaccine administered PO, ID, IN, or SQ induces protective immunity against respiratory challenge with SARS-CoV-2.

### MN vaccine administered PO, ID, SQ, and IN induces TH1-biased antibody against the N protein in hamsters after one, two, or three immunizations.

**FIG 6:**
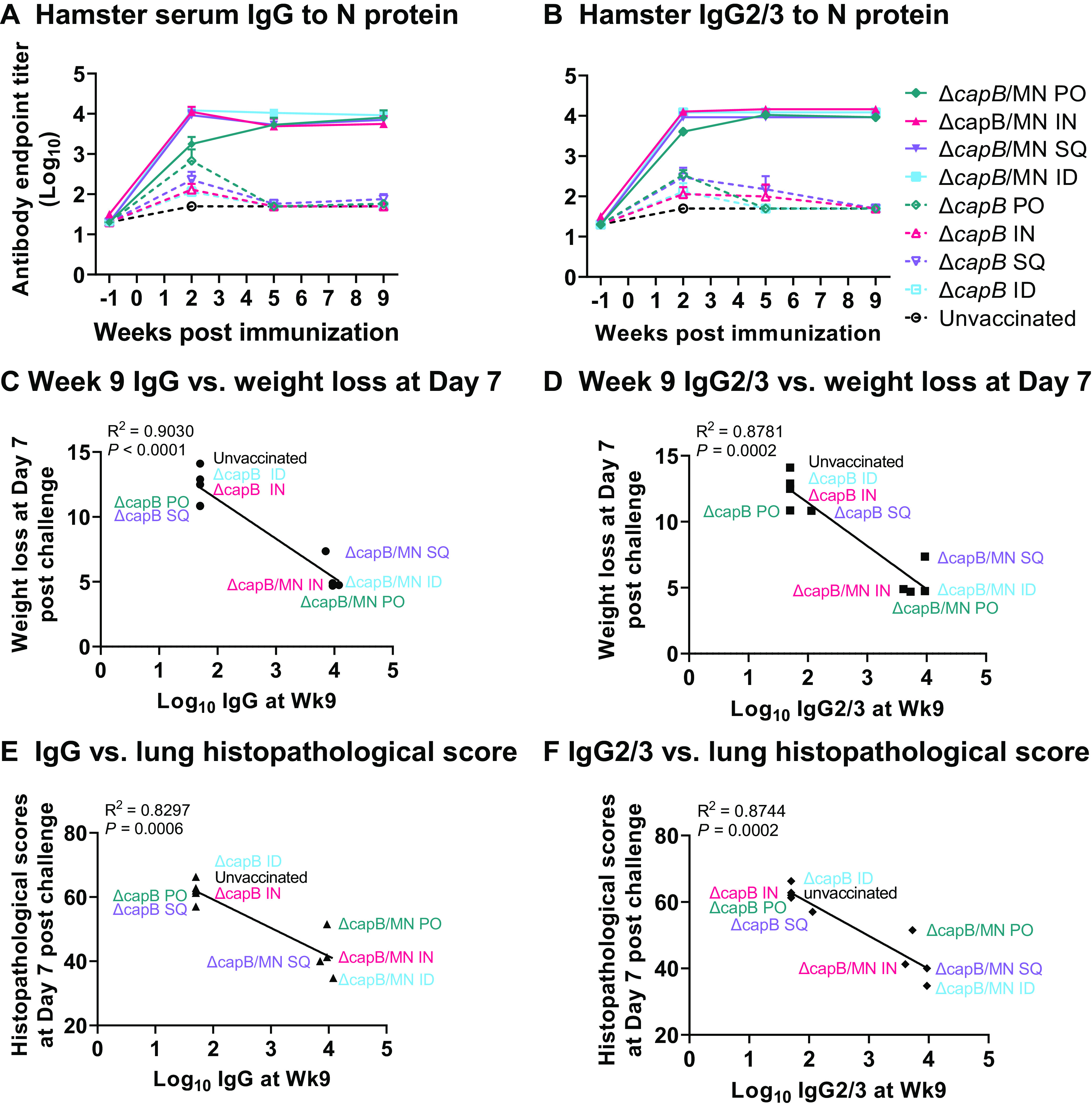
Oral administration of an rLVS Δ*capB*/MN vaccine induces serum antibody response to nucleocapsid (N) antigen that correlates with protection. Syrian hamsters (8/group, 4 female, 4 male) were immunized and challenged as described in Fig. 2A. At 1 week prior to each immunization and challenge (weeks −1, 2, 5, and 9), animals were bled and their sera tested for antibody. (A and B) Serum antibody IgG (A) and IgG2/3 (B). Values are mean ± SE. Solid lines and closed symbols represent the MN vaccine groups; dashed lines and open symbols represent the LVS Δ*capB* vector and unvaccinated control groups. Differences in antibody titer between MN vaccinated and unvaccinated animals were statistically significant for both IgG and IgG2/3 at weeks 2, 5, and 9 (*P* < 0.0001 by two-way ANOVA with Tukey’s multiple comparisons test, GraphPad Prism 9.2.0). (C and D) Correlation between pre-challenge (week 9) serum log_10_ IgG (C) and Log_10_ IgG2/3 (D) endpoint titers and weight loss on day 7 post-challenge. (E and F) Correlation between week 9 log_10_ IgG (E) and log_10_ IgG2/3 (F) endpoint titers and histopathological scores on day 7 post-challenge. Data in panels C to F were analyzed by simple linear regression test (GraphPad Prism 9.2.0).

We also analyzed the antibody response induced by the MN vaccine administered PO in hamsters. While the N protein-specific serum IgG antibody remained at the baseline level 1 week prior to immunization (Fig. S4), hamsters immunized PO three times with the MN vaccine produced significantly elevated levels of N protein-specific serum antibody on week 2 (Fig. 6A, Fig. S5A), which increased slightly and plateaued on weeks 5 (Fig. S5B) and 9 (Fig. S5C) (*P* < 0.0001 vs. unvaccinated animals at all three time points); the antibody response induced by the MN vaccine administered PO was comparable to that induced by the vaccine administered ID, SQ, and IN. In contrast, as expected, hamsters immunized with the LVS Δ*capB* vector did not produce significantly elevated levels of N-protein specific antibody after week 2. As previously observed, the serum antibody response was TH1-biased, dominated by the IgG2/3 subtype (Fig. 6B, Fig. S5). Anti-N IgG1 subtype antibody remained at baseline levels for all groups throughout the experiment (Fig. S5).

### MN vaccine-induced protection against weight loss and histopathology is correlated with anti-N antibody.

We assessed the correlation coefficient between serum N protein-specific IgG and IgG2/3 antibody endpoint titers just before challenge on week 9 and weight loss on day 7 post-challenge by linear regression analysis. N protein-specific antibody IgG and IgG2/3 endpoint titers were significantly inversely correlated with weight loss (*P < *0.0001 and *P = *0.0002 for IgG and IgG2/3, respectively) and the IgG and IgG2/3 endpoint titers were also significantly inversely correlated with lung histopathological scores on day 7 (*P = *0.0006 and *P = *0.0002 for IgG and IgG2/3, respectively) as shown in Fig. 6C–F.

## DISCUSSION

Previously, we demonstrated that the rLVS Δ*capB*/MN vaccine expressing the SARS-CoV-2 M and N proteins induces protective immunity against COVID-19-like disease in the demanding golden Syrian hamster model when administered ID or IN. Here, we significantly extended these studies by demonstrating the efficacy of the vaccine administered orally. The orally administered vaccine protected against severe weight loss, and protection was at least as strong as when administered ID, IN, or SQ. The orally administered vaccine also significantly protected against lung pathology and significantly reduced the viral load in the oropharynx and lungs.

Oral administration of the MN vaccine was efficacious despite the suboptimal methodology for delivering such a highly acid-sensitive vaccine by this route. Although F. tularensis can infect by the oral route, the infectious dose is very high, with an oral 50% infective dose [ID_50_] of 10^8^ in humans challenged with virulent *F. tularensis* subsp. *tularensis* (SchuS4) (24), consistent with its poor survival at pH 2.5 in synthetic gastric fluid replicating the fasting human stomach (25). LVS, from which our vaccine vector is derived, is even more sensitive to such acidic conditions than its parental wild-type F. tularensis, with <0.001% survival after 120 min (25). Hence, measures had to be taken to protect the MN vaccine from acid conditions in the stomach. Ideally, the MN vaccine would have been administered in a capsule designed to protect it from stomach acid and enable it to be released in the intestine. In the absence of the availability of such technology for administering the vaccine to small animals, we instead opted to deliver the vaccine shortly after administering of a dose of sodium bicarbonate to neutralize stomach acid, an approach used in studies of other vaccines administered by the oral route (26, 27). To further compensate for the suboptimal administration, we administered the vaccine on a Monday-Wednesday-Friday schedule, i.e., three times instead of once at each week of administration, because a preliminary study in mice showed that this regimen somewhat enhanced the immunogenicity of the vaccine (Fig. 1). In future studies in humans, the vaccine will presumably be administered in an acid-resistant capsule, as in the case of the oral typhoid vaccine Vivotif (Typhoid Vaccine Live Oral Ty21a) (28), which is similarly acid-sensitive.

None of the COVID-19 vaccines currently approved for human use are suitable for oral administration, and we are aware of only two which have demonstrated protective efficacy in animal models after oral administration: an enveloped virus-like particle expressing the S ± M proteins (29) and an adenovirus type 5-vectored vaccine expressing the S protein (30, 31).

In our previous study of the MN vaccine, two immunizations of the vaccine ID or IN, 4 weeks apart, were sufficient to induce strong protective immunity in the hamster model. Here, given the challenge of administering our highly acid-sensitive vaccine orally, we employed three immunizations to enhance the likelihood of successful oral administration. The fact that oral administration was comparable to or better than administration by the ID or IN routes suggests that two immunizations may similarly be sufficient to induce strong protective immunity by the oral route under optimal conditions for vaccine delivery, e.g., employing a capsule to bypass stomach acid.

Although our vaccine protected very well against severe weight loss and lung pathology, as with other vaccines, it was not fully protective in the hamster model after a high-dose respiratory challenge with SARS-CoV-2. For example, in our study, hamsters vaccinated PO with the MN vaccine and challenged 4 weeks later intranasally with the highly virulent SARS-CoV-2 strain 2019-nCoV/USA-WA1/2020 had 7% maximum weight loss (day 4) over 7 days post-challenge versus 14% (day 7) for unvaccinated hamsters. In a study by Langel et al. (30), hamsters vaccinated with an oral adenovirus vaccine expressing the S protein and challenged 3 weeks later intranasally with the same strain, as in our study, showed ~4% maximum weight loss (day 2 to 5) over 5 days post-challenge versus ~11% (day 5) for mock-vaccinated hamsters. In a study by Hajnik et al. (32), hamsters vaccinated intramuscularly with a combination of mRNA vaccines expressing the S and the N proteins (mRNA-S+N) and challenged 2 weeks later with a SARS-CoV-2 Delta strain had ~3% maximum weight loss (day 2) over 4 days post-challenge compared with ~5% (day 4) for the mock-vaccinated hamsters.

As noted previously, the MN vaccine is a potential universal COVID-19 vaccine because it is based on immunity induced by the highly conserved M and N proteins. We opted to evaluate the efficacy of oral administration of the vaccine against the 2019-nCoV/USA-WA1/2020 strain, a CDC reference strain which was originally recovered in January 2020 from a person who returned to Washington state from China and is widely used by research laboratories (33), rather than a more recent but less virulent strain. Given the high homology of the M and N proteins from this strain and currently circulating Omicron variants, the vaccine is anticipated to protect well against these later emerging variants, although this remains to be demonstrated.

The availability of a potentially universal COVID-19 vaccine that can be administered orally could be a game-changer in the evolving COVID-19 pandemic. In resource-poor countries, oral administration would circumvent the shortage of needles, syringes, and trained personnel needed to administer an injectable vaccine. Indeed, the availability of an oral vaccine that can be self-administered could simplify vaccine distribution everywhere. Moreover, an oral vaccine would eliminate one cause of vaccine hesitancy everywhere—the fear of needles. In addition, the availability of a vaccine to universally protect against COVID-19 would eliminate the need for repeated administration of newly designed vaccines against newly emerging variants.

In addition to being a potential universal vaccine capable of oral immunization, the MN vaccine has additional major advantages. (i) High antigen delivery: the intracellular bacterial vector disseminates widely in the host and, due to its ultra-high plasmid copy number (mean 171/genome), expresses exceptionally abundant recombinant protein. (ii) Low cost of large-scale manufacture: as a replicating bacterium-vectored vaccine, the vaccine can be manufactured at large scale by growth overnight in simple broth culture without the need for extensive purification as in the case of RNA vaccines, protein/adjuvant vaccines, or viral-vectored vaccines grown in mammalian culture. Moreover, there is no need for an expensive adjuvant. (iii) Ease of storage and transport: after lyophilization, the MN vaccine should be able to be stored and transported at refrigerator temperature. (iv) Safety: previous human trials have demonstrated acceptable safety of the double-deletional parent vaccine (LVS). The much more attenuated but still highly immunogenic triple-deletional platform vector (LVS Δ*capB*) derived from the parent vaccine is >10,000-fold less virulent than LVS in a mouse model (as measured by intranasal LD_50_; all animals survived the highest dose tested). Hence, the vaccine should be exceptionally safe.

We did not test efficacy of the vaccine against the currently dominant Omicron strain because of the low virulence of this strain in golden Syrian hamsters. In a study by Yuan et al. (34) comparing the Delta and Omicron variants, hamsters challenged with Omicron barely lost weight in the 7 days post-challenge, whereas hamsters challenged with Delta lost ~10% of their total body weight (*P < *0.001 versus hamsters challenged with Omicron). In our study using the wild type 2019-nCoV/USA-WA1/2020 strain, the unvaccinated hamsters lost 14% of their total body weight by day 7 post-challenge. Also, in the Yuan et al. study, Omicron-challenged hamsters had significantly lower clinical scores of illness than Delta-challenged hamsters at both 4 and 7 days post-challenge and clinical scores on day 7 were at or near zero; consistent with this, histopathology in Omicron-challenged animals was near normal at day 7, when pathology peaked in our studies. Finally, Omicron-challenged animals had >1-log fewer PFU/g in their nasal turbinates and lungs on day 4 post-challenge than Delta-challenged animals (*P < *0.0001) (34). These results are consistent with the findings of Meng et al. (35) showing much lower replication of the Omicron variant than the Delta variant in human lung and gut cells. Hence, disease caused by the Omicron strain in hamsters is so mild that it would be difficult to assess the protective effect of vaccination.

Vaccine efficacy was highly correlated with anti-N antibody, which was predominantly TH1-biased IgG2/3. Anti-N IgG and IgG2/3 antibody was highly correlated with protection against weight loss and lung histopathology. Protection is likely mediated by both humoral and cell-mediated immunity. With respect to the potential role of antibody, as described above, a recent study found that the N protein is displayed on the surface of SARS-CoV-2-infected cells and that these cells are a potential target for ADCC (17). With respect to cell-mediated immunity, it is noteworthy that the N protein is far and away the dominant SARS-CoV-2 protein recognized by CD8^+^ T cells of COVID-19 convalescent patients, recognized by 57% of these patients, and among patients with SARS-CoV-2 responding CD8^+^ T cells, a median of 43% of their CD8^+^ T cells recognized N peptides (36).

In summary, the rLVS Δ*capB*/MN vaccine, a potential universal vaccine against COVID-19, is efficacious against COVID-19-like disease when administered orally in a highly demanding animal model. This conveniently administered, easily manufactured, inexpensive, and readily stored and transported vaccine could play a major role in ending the COVID-19 pandemic by protecting immunized individuals from serious disease from current and future strains of SARS-CoV-2.

## MATERIALS AND METHODS

### Ethics statement.

Mice and hamsters were used according to protocols approved by the Institutional Animal Care and Use Committees of the University of California Los Angeles (UCLA) and Colorado State University (CSU), respectively.

### Virus and bacterium-vectored vaccines.

We acquired the SARS-CoV-2 virus (2019-nCoV/USA-WA1/2020 strain) through the NIH NIAID Biodefense and Emerging Infections Research Resources Repository (BEI Resources, NR-52281, lot no. 700033175), passaged three times in Vero E6 cells, prepared frozen stocks in DMEM supplemented with 10% fetal bovine serum, and determined the virus titer by plaque assay as we described previously (6). F. tularensis live vaccine strain with a deletion in *capB* (LVS Δ*capB*), and recombinant LVS Δ*capB* expressing the fusion protein of SARS-CoV-2 membrane and nucleocapsid proteins from a shuttle plasmid (rLVS Δ*capB*/MN) or an antigen expression cassette, along with other essential elements integrated at the deleted *capB* locus (rLVS Δ*capB*::MN), were constructed and characterized as we described previously (6, 7). Stocks of LVS Δ*capB* vector, rLVS Δ*capB*/MN, and rLVS Δ*capB*::MN vaccines were prepared in broth medium (6).

### Proteins, peptides, and heat-inactivated bacteria.

We obtained the following reagents through BEI Resources: SARS-CoV-2 nucleocapsid protein with an N-terminal histidine tag prepared from recombinant E. coli (NR-53246), SARS-CoV-2 N protein peptide array (NR-52404), and SARS-CoV membrane protein with a C-terminal histidine tag prepared from recombinant E. coli (NR-878). Stocks of heat-inactivated LVS Δ*capB* (HI-LVS) were prepared as described previously (7).

### Optimization of oral vaccination in mice.

Six to 8-week-old specific-pathogen-free female BALB/c mice were purchased from Jackson Laboratories (Bar Harbor, ME.). The mice were immunized orally (PO) by gavage once (Monday [M]) or three times a week (Monday, Wednesday, Friday [MWF]) on weeks 0 and 3 with three escalating doses (10^6^, 10^7^, and 10^8^, diluted in 0.2 mL NS) of the rLVS Δ*capB*::MN vaccine. Mice immunized PO once (M) a week with 0.2 mL NS or three times (MWF) a week with 10^8^ LVS Δ*capB* vector, and mice immunized once a week intradermally (4 × 10^6^ CFU in 0.05 mL NS), intranasally (1 × 10^6^ CFU in 0.02 mL NS), or subcutaneously (4 × 10^6^ CFU in 0.05 mL NS) with the rLVS Δ*capB*::MN vaccine at week 0 and week 3 served as controls. Thirty minutes prior to PO immunization, the animals were given 0.1 mL of 10% (wt/vol) sodium bicarbonate by gavage to neutralize gastric acid. Two weeks after the second immunization, mice were anesthetized by an intraperitoneal injection of ketamine (10 mg/mL)-xylazine (1 mg/mL) solution, bled, and subsequently euthanized by inhalation of CO_2_. Sera were isolated, heat-inactivated, and frozen at −80°C until use. Mouse spleens and lungs were removed, and single cell suspensions of spleen and lung cells prepared as described previously (6, 37, 38). Antigen-specific serum antibody and T cell-mediated immune responses were examined as published previously (6) and described below.

### Enzyme-linked immunosorbent assay for assaying antibodies in mouse sera.

Mouse sera were assayed for IgG to HI-LVS antigens as published previously (6). Briefly, high-binding 96-well plates (Costar) were coated with 0.1 mL HI-LVS (pre-heat-inactivation titer of 5 × 10^6^/mL) diluted in carbonate/bicarbonate buffer for overnight at 4°C and blocked with Blocker Casein in phosphate-buffered saline (PBS; Thermo Fisher Scientific, Waltham, MA) for 1 h at ambient temperature. Sera at a starting dilution of 1:20 in 1% bovine serum albumin (BSA)-PBS were diluted 6 times further through a 3-fold series and incubated with HI-LVS antigens coated on 96-well plates overnight at 4°C. Afterwards, the wells were incubated for 90 min with alkaline phosphatase (AP)-conjugated goat anti-mouse IgG (Sigma-Aldrich, St. Louis, MO) at a dilution of 1:1,000 at ambient temperature, followed by incubation for 20 min with 100 μL of NPP (*p*-nitrophenylphosphate) substrate in diethanolamine buffer (Phosphatase Substrate kit, Bio-Rad, Hercules, CA). The plates were thoroughly washed 3 to 4 times with PBS supplemented with 0.05% Tween 20 between each reaction. The reaction was stopped by adding 100 μL of 0.1 N sodium hydroxide and the solutions were read at 415 nm for absorbance.

### *In vitro* stimulation and production of IFN-γ by murine immune splenocytes and lung cells.

Mouse splenocytes and lung cells were stimulated with antigens and assayed for production of IFN-γ as we published previously (6). Briefly, a single cell suspension of 1.0 × 10^5^ splenocytes or lung cells per well was seeded in U-bottom 96-well plates and incubated with T cell medium (Complete Advanced RPMI 1640 [Invitrogen] supplemented with 1 mM HEPES [Cellgro, Mediatech], 2 mM l-alanyl-l-glutamine [GlutaMAX Supplement, Gibco], 50 μM 2-mercaptoethanol [Sigma-Aldrich], 100 U/mL penicillin/100 μg/mL streptomycin, and 2% fetal bovine serum) alone or T cell medium supplemented with 2 μg/mL of N protein, 2 μg/mL of N peptide pool, or 2 μg/mL of M protein of SARS-CoV-2 or 5 × 10^6^ HI-LVS for 3 or 6 days. Afterwards, the culture supernatant fluid was collected, cell debris removed by centrifugation, and the supernatant fluid diluted 5-fold and stored in assay diluent (BD Biosciences) at −80°C until use. The production of mouse IFN-γ in the culture supernatant fluid was assayed using a mouse cytokine EIA kit (BD Biosciences) per the manufacturer’s instructions.

### Efficacy study in hamsters.

Golden Syrian hamsters (Mesocricetus auratus), 9 weeks old, were purchased from Charles River Laboratories (Wilmington, MA). Animals (8/group; 4 males, 4 females) were immunized intradermally, subcutaneously, intranasally, or orally three times, 3 weeks apart (weeks 0, 3, and 6), with 4 × 10^6^ CFU ID or SQ, 2 × 10^6^ CFU IN, or 1 × 10^9^ CFU PO of LVS Δ*capB* vector or rLVS Δ*capB*/MN vaccines diluted in 0.1 mL (ID and SQ), 0.02 mL (IN), or 0.2 mL (PO) sterile PBS; challenged IN 4 weeks later on week 10 with 10^4^ PFU of SARS-CoV-2 (2019-nCoV/USA-WA1/2020 strain) in a volume of 100 μL; and monitored closely for clinical signs of infection including weight loss, as we published previously (6); animals were also monitored for level of activity, nasal discharge, and response to simulation. Thirty minutes prior to each oral immunization, hamsters were given 0.5 mL of 1% sodium bicarbonate by gavage to protect the bacterium-vectored vaccine from gastric acid. Unvaccinated hamsters served as controls. Animals immunized ID, IN, or SQ were immunized only on Monday of each vaccination week; animals immunized PO were immunized Monday, Wednesday, and Friday of each vaccination week to compensate for suboptimal oral administration in small animals. Blood was collected 4 to 8 days prior to each immunization and challenge to assess antibody responses, and the sera were heat-inactivated at 56°C for 30 min. Hamsters were moved to an animal biosafety level 3 facility (ABSL-3) 6 days prior to challenge. Animals (*n* = 8/group) were monitored daily post-challenge for clinical signs of infection (fever, weight loss, nasal discharge, etc.) and weighed daily on days 0 to 7 post-challenge, and the oropharynx was swabbed for virus titers on days 1, 2, and 3 post-challenge. Half of the animals (4 hamsters, 2 females, 2 males) in each group were euthanized on day 3 post-challenge (acute phase) to assess lung (cranial and caudal lobes) virus titers, which peak on day 3, and the other half (4 hamsters, 2 females, 2 males) of each group were euthanized on day 7 (subacute phase) post-challenge to evaluate lung histopathology, which peaks at that time.

### Histopathology assessment.

Lung tissues from hamsters were fixed in 10% buffered formalin for 7 to 14 days and embedded in paraffin. Cut sections were processed, and cranial and caudal histopathological scores evaluated separately as we have described previously (6).

### Percent alveolar air space quantitation.

Alveolar air space was quantitated using Visiopharm software version 2017.2.4.3387. Briefly, hematoxylin-and-eosin-stained sections of the left caudal lung lobe from each animal were scanned at ×40 magnification using an Olympus VS120 microscope scanner, Hamamatsu ORCA-R2 camera, and Olympus VS-ASW 2.9 software at the Microscopy Core of the Translational Research Institute (TRI), Brisbane, Queensland, Australia. Regions of interest, defined as alveolar airspaces with exclusion of large to medium bronchioles and large to medium blood vessels, were selected and edited by a pathologist before quantification. Median percent air space was determined for each group.

### Virus assay.

Virus titration was performed on oropharyngeal swabs obtained at 1, 2, and 3 days post-challenge and on tissue samples of cranial and caudal lungs obtained at 3 days post-challenge by double-overlay plaque assay on Vero E6 cells as previously described (6, 39).

### Enzyme-linked immunosorbent assay for anti-SARS-CoV-2 N antibody in hamster sera.

Hamster sera were assayed for IgG and IgG2/3 subtype antibodies specific to SARS-CoV-2 N protein antigens by enzyme-linked immunosorbent assay (ELISA) as described previously (6, 7).

### Statistics.

A linear regression was used to compute the correlation (*r*) between mean N protein-specific serum IgG and IgG2/3 antibody endpoint titers pre-challenge and weight loss at day 7 and also between IgG and IgG2/3 endpoint titers and total lung (left lung and right lung cranial and caudal lobes) histopathological scores on day 7 post-challenge. Mean and standard error of the serum antibody endpoint titer are reported, and means were compared between groups by two-way analysis of variance (ANOVA) with Tukey’s correction for multiple-comparison test using GraphPad Prism version 9.2.0 (San Diego, CA). Viral titers and lung histopathological scores were compared by one-way ANOVA with Fisher’s least significant difference (LSD) multiple-comparisons test (GraphPad Prism 9.2.0). For the mouse immunology experiments, the sample sizes for assaying immune responses post-vaccination (4/group) were estimated based on previous studies with 80% power using an alpha = 0.05 (i.e., *P < *0.05) significance criterion. Mean and standard error of serum antibody endpoint titer and cytokine production are shown. Means are compared across groups by one-way or two-way ANOVA with Tukey’s correction for multiple-comparison test using GraphPad Prism 9.2.0.

### Data availability.

All data supporting our findings study are available within the article and supplemental information files or from the corresponding author upon request.
