# Supplementary material for: Oral Administration of Universal Bacterium-Vectored Nucleocapsid-Expressing COVID-19 Vaccine is Efficacious in Hamsters
Source: Microbiol Spectr. 2023 Mar 14;11(2):e05035-22. doi: 10.1128/spectrum.05035-22 (PMC10100875; doi:10.1128/spectrum.05035-22)
Supplement: Supplemental file 1 — Fig. S1 to S5 and Table S1. Download spectrum.05035-22-s0001.pdf, PDF file, 2.8 MB [file spectrum.05035-22-s0001.pdf]

Supplemental Materials

Supplemental Figures

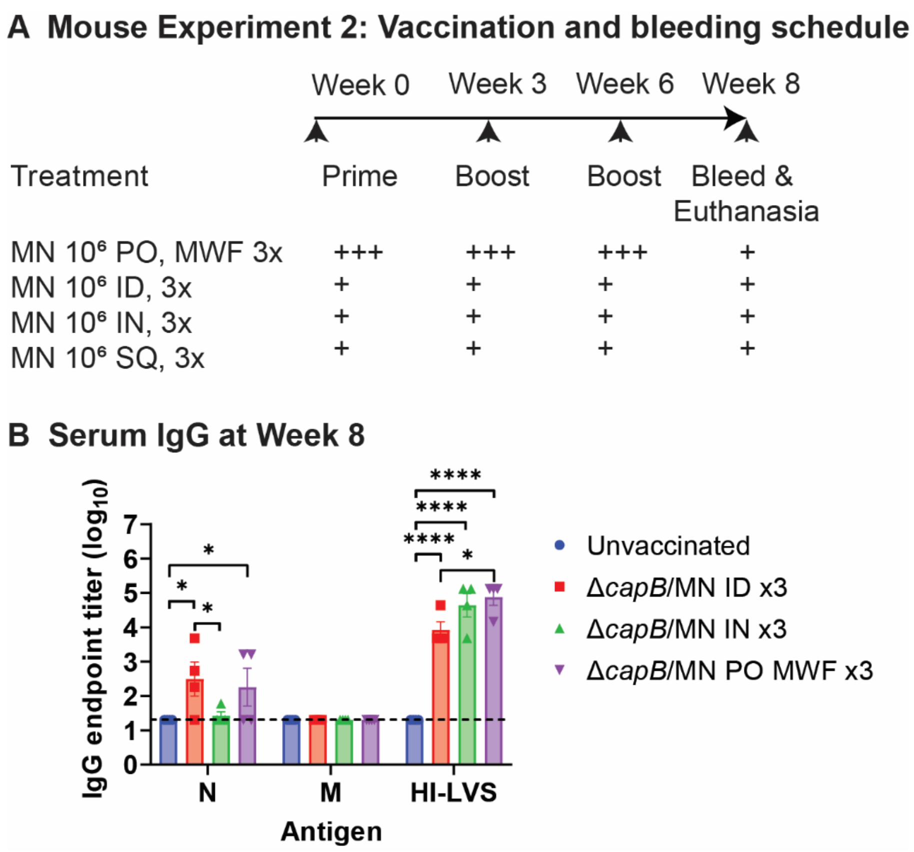

Figure S1. Oral administration of the rLVS Δ*capB*/MN vaccine to mice induces antibody to nucleocapsid protein at levels comparable to intradermal and intranasal administration.

A. Experiment schedule**.** BALB/c mice, 4/group, were unvaccinated (Unvaccinated) or vaccinated with the rLVS Δ*capB*/MN vaccine (Δ*capB*/MN) intradermally (ID) with 4 x 10^6 CFU or intranasally (IN) with 10^6 CFU at Week 0, 3, and 6 on Mondays, or orally (PO) with 10^9 CFU at Week 0, 3, and 6 on Mondays, Wednesdays, and Fridays (MWF). At Week 8, mice were bled, euthanized, and spleens and lungs removed. **B.** Serum IgG at Week 8. Sera were assayed for IgG antibody specific to SARS-CoV-2 nucleocapsid (N) or membrane (M) protein or to heat-inactivated LVS Δ*capB* (HI-LVS). The antibody endpoint titer is expressed as Log_{10} the

reciprocal of the highest serum dilution that is a minimum of 0.05 optical density units above the Mean of the unvaccinated mice control serum plus 3 standard deviations at the same dilution. *, $p < 0.05$; ****, $p < 0.0001$ by 2-WAY ANOVA with Holm-Šídák's multiple comparisons test (GraphPad Prism 9.2.0).

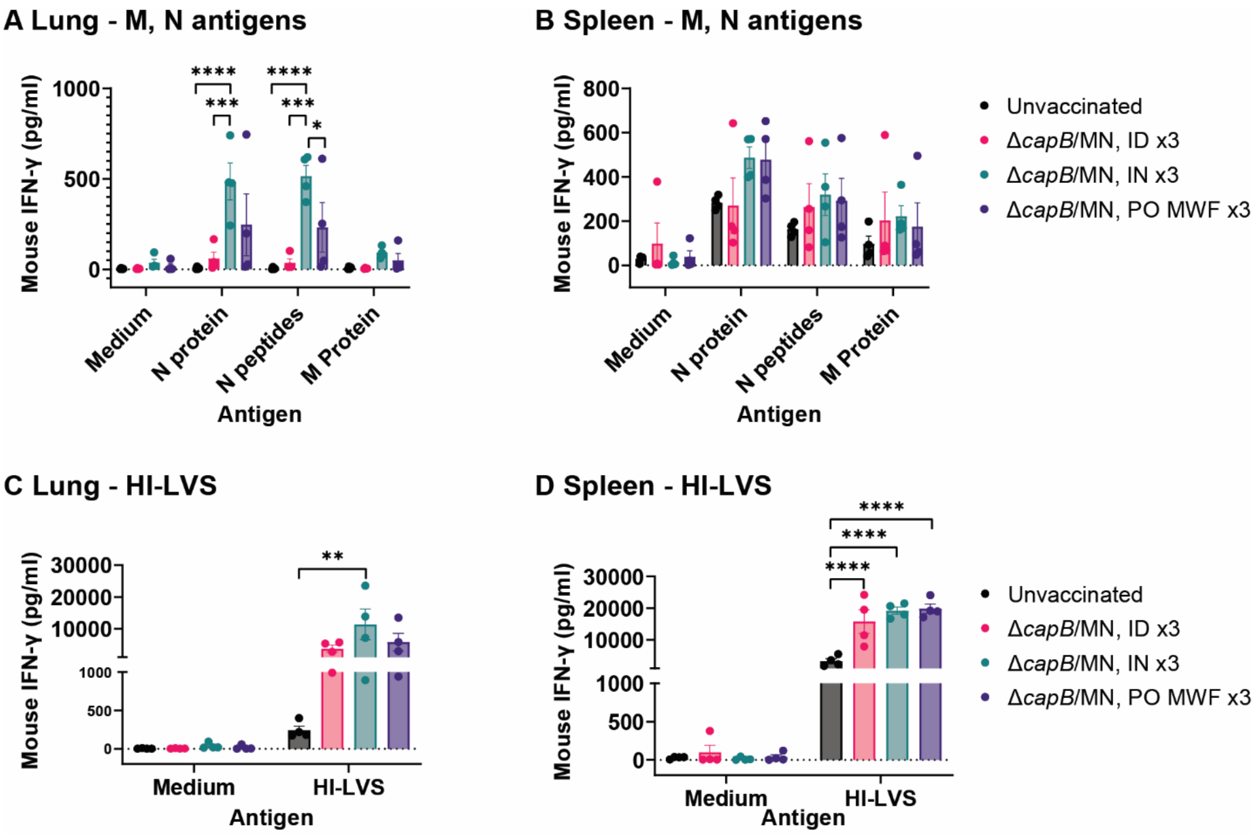

Figure S2. Oral administration of the rLVS Δ*capB*/MN vaccine to mice induces IFN-γ secretion in response to nucleocapsid protein/peptides and HI-LVS stimulation. BALB/c mice, 4/group, were unvaccinated (Unvaccinated) or vaccinated ID, IN or PO with the rLVS Δ*capB*/MN vaccine (Δ*capB*/MN) and bled, euthanized, and their spleens and lungs removed at Week 8 as described in the legend to Figure S1A. Lung and spleen cells were incubated in Medium-T without antigen (Medium), or with 2 µg/ml of Nucleocapsid protein (N protein), N peptide pool (N peptides), or Membrane protein (M protein), or with heat-inactivated LVS (HI-LVS) at 37°C for 3 days. After 3-days stimulation, the culture supernatant fluid was collected and assayed for IFN-γ production. **A and B.** Lung (A) and spleen (B) cells were stimulated with T-cell medium without antigen, or N protein, N peptide pool, or M protein for 3 days. **C and D.** Lung (C) and spleen (D) cells were stimulated with T-cell medium without antigen or with HI-

LVS for 3 days. *, $p < 0.05$; ***, $p < 0.001$; and ****, $p < 0.0001$ by 2-WAY ANOVA with Holm-Šídák's multiple comparisons test (GraphPad Prism 9.2.0).

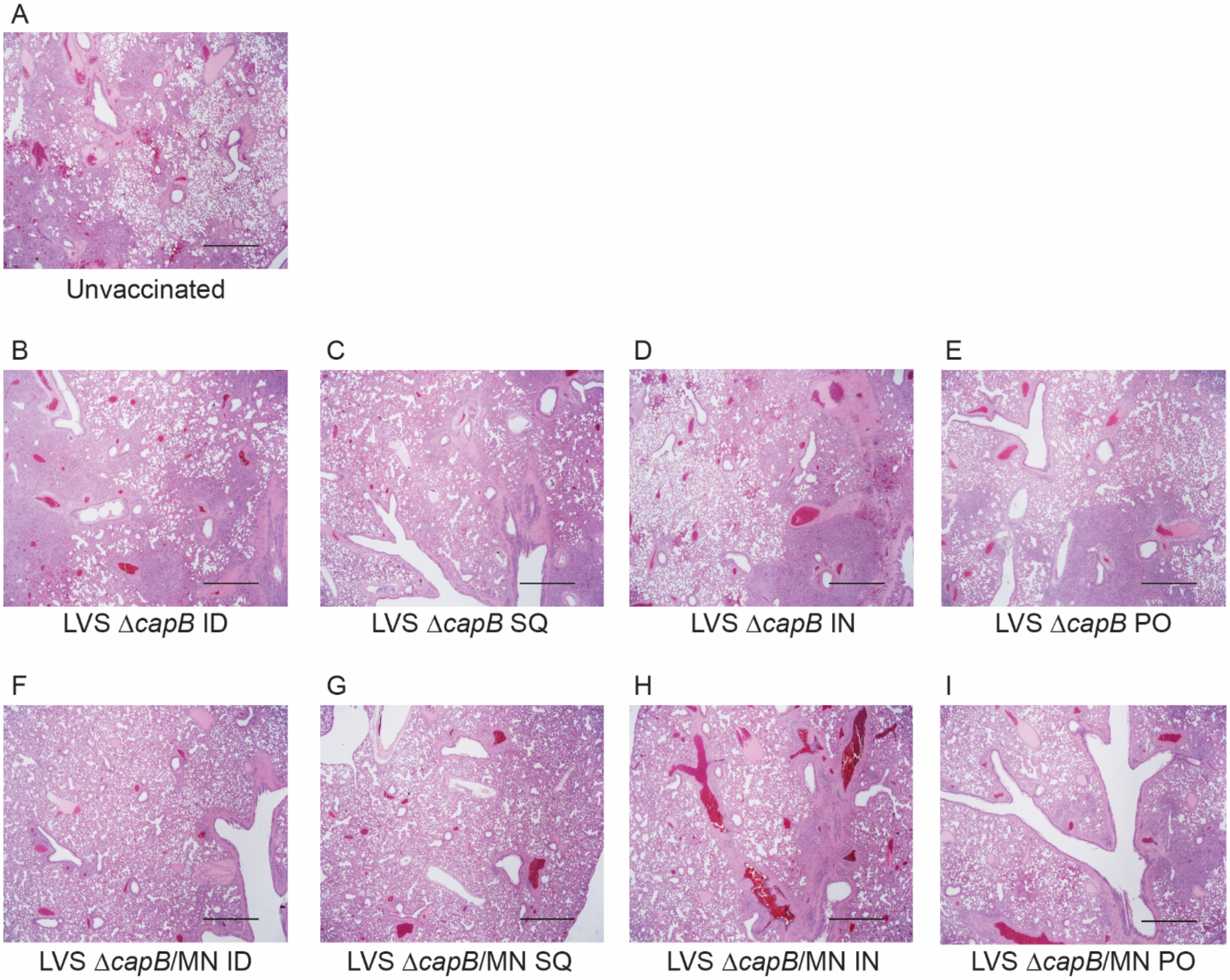

Figure S3. Histopathological changes in the lung. Hamsters were unvaccinated (A) or vaccinated ID, SQ, IN, or PO with LVS Δ*capB* vector (B, C, D, and E, respectively) or with rLVS Δ*capB*/MN (F, G, H, and I, respectively), challenged IN with SARS-CoV-2, and their lungs removed and assessed for histopathological changes at 7 days post infection, as indicated in **Fig**. **1A**. Lung tissues were fixed in 10% buffered formalin and embedded in paraffin, and lung sections were cut and stained with hematoxylin and eosin. The microphotos shown are from animals that were closest to the group average. (Scale bar: 800 μm).

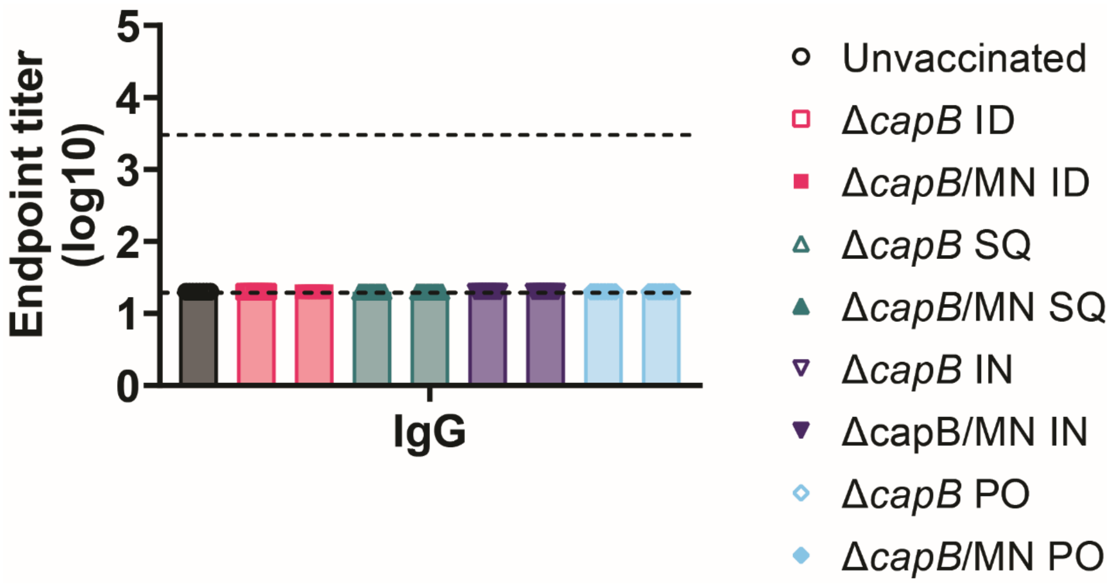

Figure S4. Basal level of anti-N serum antibody in hamsters. Syrian hamsters (8/group, 4F, 4M) were immunized and challenged as described in Figure 2A. At one week prior to immunization, animals were bled and their sera tested for anti-N IgG antibody. Values are mean ± SE. Open symbols represent the LVS Δ*capB* vector and unvaccinated control groups; closed symbols represent the MN vaccine groups. Top and bottom dashed lines represent maximum and minimum detection limit.

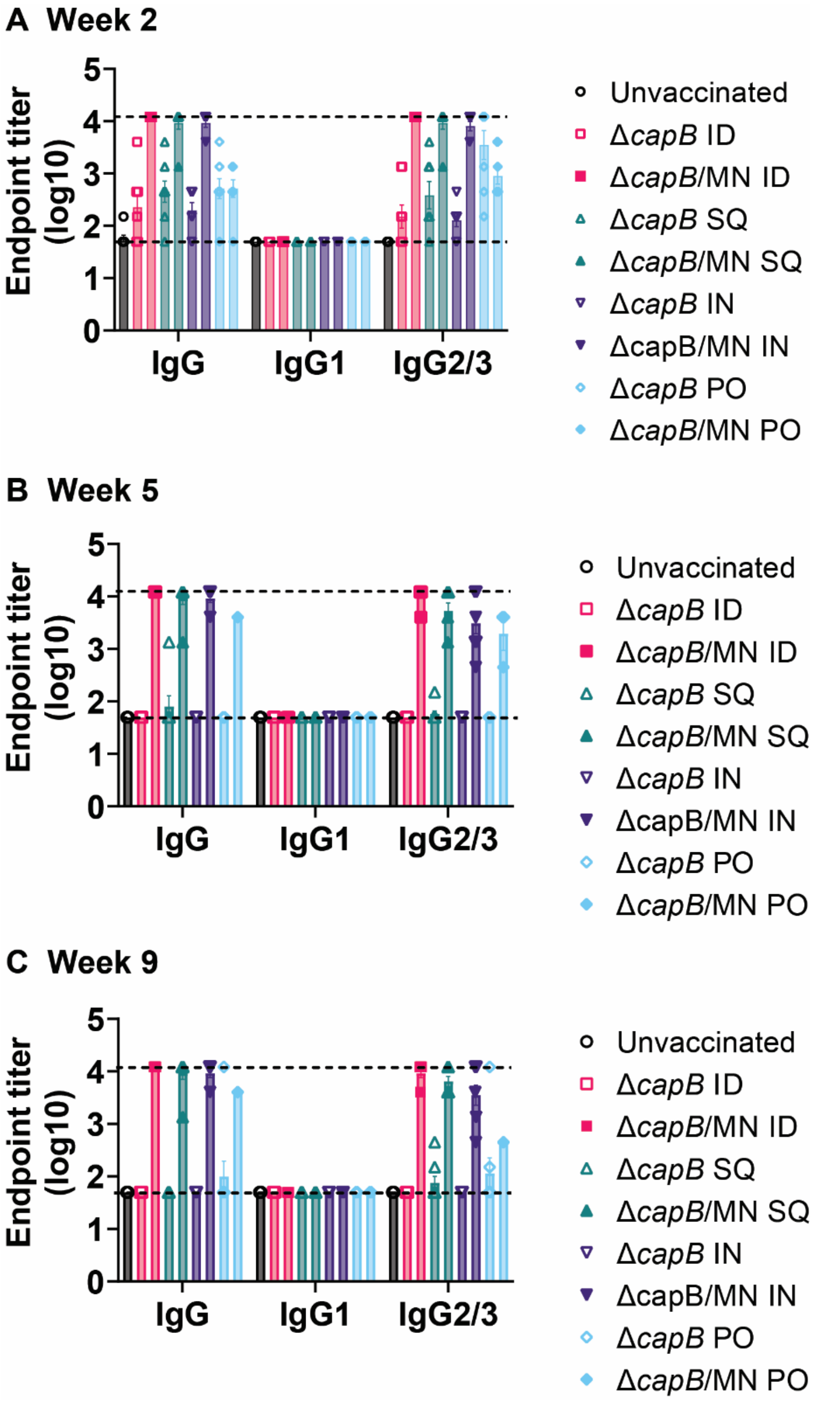

Figure S5. Oral administration of an rLVS Δ*capB*/MN vaccine induces serum antibody response to nucleocapsid (N) antigen that correlates with protection. Syrian hamsters (8/group, 4F, 4M) were immunized and challenged as described in Figure 2A. At one week prior to each immunization and challenge, Weeks -1 (see Fig. S4), 2 (A), 5 (B) and 9 (C), animals

were bled and their sera tested for antibody IgG and subtype IgG2/3 (favoring a Th1 response in hamsters) and IgG1 (favoring a Th2 type response in hamsters). Values are mean ± SE. Open symbols represent the LVS Δ*capB* vector and unvaccinated control groups; closed symbols represent the MN vaccine groups. Top and bottom dashed lines represent maximum and minimum detection limit. Data for IgG and IgG2/3 are also presented in Figure 6.

Supplemental Table

Table S1. Lung histopathological scores at Day 7 post challenge

Animal #	Tissue	Overall lesion extent	Bronchitis	Alveolitis	Pneumocyte hyperplasia	Vasculitis	Interstitial inflammation	Total score	Total lung score
**A-3**	**Left lung**	3	3	4	4	3	4	21	66
	**Right cranial lobe**	4	3	4	4	3	4	22	
	**Right caudal lobe**	4	3	5	4	3	4	23	
**A-4**	**Left lung**	3	3	4	4	3	3	20	64
	**Right cranial lobe**	3	3	4	4	4	4	22	
	**Right caudal lobe**	3	4	4	4	3	4	22	
**A-7**	**Left lung**	3	3	4	4	3	3	19	59
	**Right cranial lobe**	3	3	4	4	3	4	20	
	**Right caudal lobe**	3	3	4	4	3	4	20	
**A-8**	**Left lung**	4	2	4	4	3	3	20	62
	**Right cranial lobe**	4	3	4	4	3	4	22	
	**Right caudal lobe**	3	3	4	4	3	3	20	
**B-3**	**Left lung**	3	3	4	3	4	3	20	65
	**Right cranial lobe**	4	3	5	4	3	4	23	
	**Right caudal lobe**	3	3	4	4	4	4	22	
**B-4**	**Left lung**	4	3	5	4	3	4	23	70
	**Right cranial lobe**	4	3	5	5	3	4	24	
	**Right caudal lobe**	4	3	5	4	3	4	23	
**B-7**	**Left lung**	3	3	4	4	3	3	20	66
	**Right cranial lobe**	4	3	5	4	3	3	22	
	**Right caudal lobe**	4	4	5	4	3	4	24	
**B-8**	**Left lung**	3	3	4	4	3	4	21	64
	**Right cranial lobe**	2	3	3	3	4	4	19	
	**Right caudal lobe**	4	4	5	4	3	4	24	
**C-3**	**Left lung**	3	3	4	4	3	3	20	64
	**Right cranial lobe**	3	3	4	4	3	3	20	
	**Right caudal lobe**	3	4	5	4	4	4	24	
**C-4**	**Left lung**	3	2	4	4	2	3	18	41
	**Right cranial lobe**	3	3	4	4	3	4	21	
	**Right caudal lobe**	3	3	4	4	4	4	22	
**C-7**	**Left lung**	3	2	4	4	3	4	20	56
	**Right cranial lobe**	3	2	3	3	2	3	16	
	**Right caudal lobe**	3	3	4	4	2	4	20	
**C-8**	**Left lung**	4	3	5	5	2	4	23	67
	**Right cranial lobe**	4	3	5	4	3	4	23	
	**Right caudal lobe**	3	3	4	4	4	3	21	
**D-3**	**Left lung**	4	3	4	4	3	4	22	62
	**Right cranial lobe**	3	2	4	3	3	4	19	
	**Right caudal lobe**	4	3	4	3	3	4	21	
**D-4**	**Left lung**	4	3	4	4	4	4	23	68
	**Right cranial lobe**	3	3	4	4	4	4	22	
	**Right caudal lobe**	4	3	4	4	4	4	23	

Supplemental Table 1. Lung histopathological scores at Day 7 post challenge - continued

Animal #	Tissue	Overall lesion extent	Bronchitis	Alveolitis	Pneumocyte hyperplasia	Vasculitis	Interstitial inflammation	Total score	Total lung score
**D-7**	**Left lung**	3	2	3	2	3	3	16	65
	**Right cranial lobe**	4	3	5	5	2	5	25	
	**Right caudal lobe**	4	3	5	5	3	4	24	
**D-8**	**Left lung**	3	3	4	4	3	3	20	53
	**Right cranial lobe**	2	2	2	0	2	2	10	
	**Right caudal lobe**	4	3	5	4	4	3	23	
**E-3**	**Left lung**	3	3	4	4	3	3	20	60
	**Right cranial lobe**	3	2	4	3	3	4	19	
	**Right caudal lobe**	3	3	4	4	3	4	21	
**E-4**	**Left lung**	3	3	4	4	3	3	20	61
	**Right cranial lobe**	3	2	4	4	3	4	20	
	**Right caudal lobe**	3	3	4	4	3	4	21	
**E-7**	**Left lung**	3	3	4	4	3	3	20	64
	**Right cranial lobe**	4	3	5	4	4	4	24	
	**Right caudal lobe**	3	3	4	4	2	4	20	
**E-8**	**Left lung**	4	2	4	3	2	3	18	60
	**Right cranial lobe**	4	2	5	4	3	3	21	
	**Right caudal lobe**	3	3	4	4	3	4	21	
**F-3**	**Left lung**	2	2	3	3	4	4	18	49
	**Right cranial lobe**	2	2	1	0	4	4	13	
	**Right caudal lobe**	2	2	3	3	4	4	18	
**F-4**	**Left lung**	2	2	2	1	3	2	12	32
	**Right cranial lobe**	1	1	1	0	4	3	10	
	**Right caudal lobe**	1	1	1	0	4	3	10	
**F-7**	**Left lung**	2	2	0	0	4	4	12	35
	**Right cranial lobe**	2	2	0	0	4	3	11	
	**Right caudal lobe**	2	2	0	0	4	4	12	
**F-8**	**Left lung**	1	1	2	2	2	2	10	23
	**Right cranial lobe**	1	0	0	0	2	2	5	
	**Right caudal lobe**	1	2	1	2	0	2	8	
**G-3**	**Left lung**	2	2	2	2	2	2	12	47
	**Right cranial lobe**	2	2	3	3	4	3	17	
	**Right caudal lobe**	2	2	3	3	4	4	18	
**G-4**	**Left lung**	3	1	3	3	2	2	14	33
	**Right cranial lobe**	1	2	0	0	2	1	6	
	**Right caudal lobe**	2	3	1	1	3	3	13	
**G-7**	**Left lung**	2	1	2	1	3	3	12	46
	**Right cranial lobe**	2	2	2	2	4	3	15	
	**Right caudal lobe**	2	2	3	4	4	4	19	
**G-8**	**Left lung**	2	0	2	1	3	3	11	34
	**Right cranial lobe**	2	1	0	0	3	2	8	
	**Right caudal lobe**	2	3	2	2	4	2	15	

Supplemental Table 1. Lung histopathological scores at Day 7 post challenge - continued

Animal #	Tissue	Overall lesion extent	Bronchitis	Alveolitis	Pneumocyte hyperplasia	Vasculitis	Interstitial inflammation	Total score	Total lung score
**H-3**	**Left lung**	2	0	2	0	3	3	10	45
	**Right cranial lobe**	3	3	2	2	4	3	17	
	**Right caudal lobe**	2	3	3	3	4	3	18	
**H-4**	**Left lung**	2	2	2	2	4	3	15	31
	**Right cranial lobe**	1	0	0	0	3	1	5	
	**Right caudal lobe**	2	2	0	0	4	3	11	
**H-7**	**Left lung**	2	2	2	2	4	3	15	50
	**Right cranial lobe**	3	3	1	2	4	4	17	
	**Right caudal lobe**	3	3	2	2	4	4	18	
**H-8**	**Left lung**	2	1	1	1	3	2	10	39
	**Right cranial lobe**	2	2	1	1	4	2	12	
	**Right caudal lobe**	2	3	3	3	4	3	17	
**I-3**	**Left lung**	3	0	3	2	2	2	12	51
	**Right cranial lobe**	3	2	4	4	4	3	20	
	**Right caudal lobe**	3	2	4	4	3	3	19	
**I-4**	**Left lung**	3	2	2	2	4	4	17	52
	**Right cranial lobe**	3	2	3	2	4	4	18	
	**Right caudal lobe**	2	3	2	2	4	4	17	
**I-7**	**Left lung**	3	0	1	1	4	4	13	45
	**Right cranial lobe**	3	2	3	2	4	3	17	
	**Right caudal lobe**	3	2	1	1	4	4	15	
**I-8**	**Left lung**	3	2	4	2	3	3	17	58
	**Right cranial lobe**	4	3	5	4	4	3	23	
	**Right caudal lobe**	3	3	3	3	3	3	18	
